# Effects of prey trophic mode on the gross-growth efficiency of marine copepods: the case of mixoplankton

**DOI:** 10.1038/s41598-020-69174-w

**Published:** 2020-07-23

**Authors:** Claudia Traboni, Albert Calbet, Enric Saiz

**Affiliations:** 1 Institut de Ciéncies del Mar (ICM-CSIC), Psg. Marítim Barceloneta 37-49, 08003 Barcelona, Spain; 2 Laboratoire d’Ecologie des Systèmes Aquatiques, Université Libre de Bruxelles, CP221, Boulevard du Triomphe, 1050 Brussels, Belgium

**Keywords:** Ecophysiology, Ecology, Environmental sciences, Ocean sciences, Marine biology

## Abstract

Copepod reproductive success largely depends on food quality, which also reflects the prey trophic mode. As such, modelling simulations postulate a trophic enhancement to higher trophic levels when mixotrophy is accounted in planktonic trophodynamics. Here, we tested whether photo-phagotrophic protists (mixoplankton) could enhance copepod gross-growth efficiency by nutrient upgrading mechanisms compared to obligate autotrophs and heterotrophs. To validate the hypothesis, we compared physiological rates of the copepod *Paracartia grani* under the three functional nutrition types. Ingestion and egg production rates varied depending on prey size and species, regardless of the diet. The gross-growth efficiency was variable and not significantly different across nutritional treatments, ranging from 3 to 25% in the mixoplanktonic diet compared to autotrophic (11–36%) and heterotrophic (8–38%) nutrition. Egg hatching and egestion rates were generally unaffected by diet. Overall, *P. grani* physiological rates did not differ under the tested nutrition types due to the large species-specific variation within trophic mode. However, when we focused on a single species, *Karlodinium veneficum*, tested as prey under contrasting trophic modes, the actively feeding dinoflagellate boosted the egestion rate and decreased the copepod gross-growth efficiency compared to the autotrophic ones, suggesting possible involvement of toxins in modulating trophodynamics other than stoichiometric constraints.

## Introduction

Copepods are the dominant mesozooplanktonic grazers in the marine realm, showing a wide variety of feeding modes and dietary habits^1^, and bridging unicellular producers and consumers with fish populations. Copepod feeding and reproductive performance depends on specific food traits, such as prey size^2–4^, prey concentration^5^, prey motility^6^, and food quality^7,8^. For copepods, food quality (e.g., biochemical composition and relative contribution of micro- and macronutrients of their prey^9^) is typically related to prey-type specific characteristics, ultimately associated to the prey trophic mode and nutrient availability. According to ecological stoichiometry, heterotrophic prey would benefit copepods in terms of stoichiometric balance^10^. However, from previous studies, no apparent enhancement in the egg production efficiency was observed in copepods fed heterotrophic protists compared to strict autotrophic prey^7^.

Overall, both autotrophic and heterotrophic protists make up copepod diet in different relative contributions according to the environmental nutrient regime and the particular food web type (classic herbivore vs. microbial) established^11,12^. In productive systems, such as upwellings, diatoms represent the dominant nitrogen and lipid source in copepod diets^11,13^. In oligotrophic environments, heterotrophic nano- and microplankton, such as many dinoflagellates and ciliates are the prevailing food^11^. Nevertheless, the dichotomic paradigm placing strict phototrophs and heterotrophs as major players in nutrient cycling within planktonic communities is challenged by the presence of mixotrophy. In fact, nowadays a large proportion of marine protists is acknowledged to be capable of performing both photosynthesis and phagotrophy^14^, and therefore referred herein to as mixoplankton^15^. Assigning mixoplankton to a fixed functional role in planktonic communities is a hard task due to the great variety of mixotypes existing^16^ and their flexible reliance on either nutritional strategy^15^. As a consequence, by overlooking mixoplankton as key drivers in ocean production, the predicted contribution of species to nutrient fluxes would only reflect an incomplete dichotomic food web structure^15^.

Hitherto, experimental evidences^17,18^ and model simulations^16,19^ reinforce the idea that mixoplankton do influence trophic interactions and nutrient cycling via trophic upgrade and channelling of nutrients to higher predators from unfavourably-sized prey. Nonetheless, how mixoplankton affect copepod and other zooplankton vital rates is not yet fully understood, and the outcome of the experimental evidence available is diverse. Some studies have reported either enhancement^17^ or the lack of any remarkable effect on copepod physiology when fed different potential mixotrophic dinoflagellates^20,21^, whereas when the mixoplanktonic species tested may contain toxins, as typically occur during the onset of harmful algal blooms^22–24^, other assays have showed impairment of copepod vital rates. It is worth noting, however, that mixoplankton tested in previous studies were usually not grown under phagotrophic status, rather they were reared as functional autotrophs (i.e., in inorganic nutrient-rich media and high irradiance levels without additional prey supply)^15^. Therefore, up to date, the nutritional value of functional (actively feeding) mixoplankton on copepod physiology is still under-investigated^17^ and the integration of mixoplankton as independent trophic level is only partially addressed on ecosystem scale by a few numerical models and network analyses^25,26^.

In the present study, we determined differences in the gross-growth efficiency of the calanoid copepod *Paracartia grani* (Sars) when offered functional mixotrophic prey in comparison to typically-used autotrophic and heterotrophic diets. We restricted our study to mixoplankton that do not produce (acute) toxic responses in copepods, and ensured the mixotrophic prey used in the experiments were actively feeding. To achieve this aim, we conducted grazing experiments to quantify ingestion, egg production and egg hatching rates under the aforementioned dietary conditions. We also determined the copepod fecal pellet production and fecal pellet size under the different diets tested to assess variations in egestion rate due the likely differences in the degree of prey digestion and packaging. Finally, we also explored the effects of intraspecific variability of the prey trophic mode (in both oligotrophic and eutrophic conditions) on the copepod physiological performance.

## Results

### Elemental content and stoichiometric ratios

**Figure 1:**
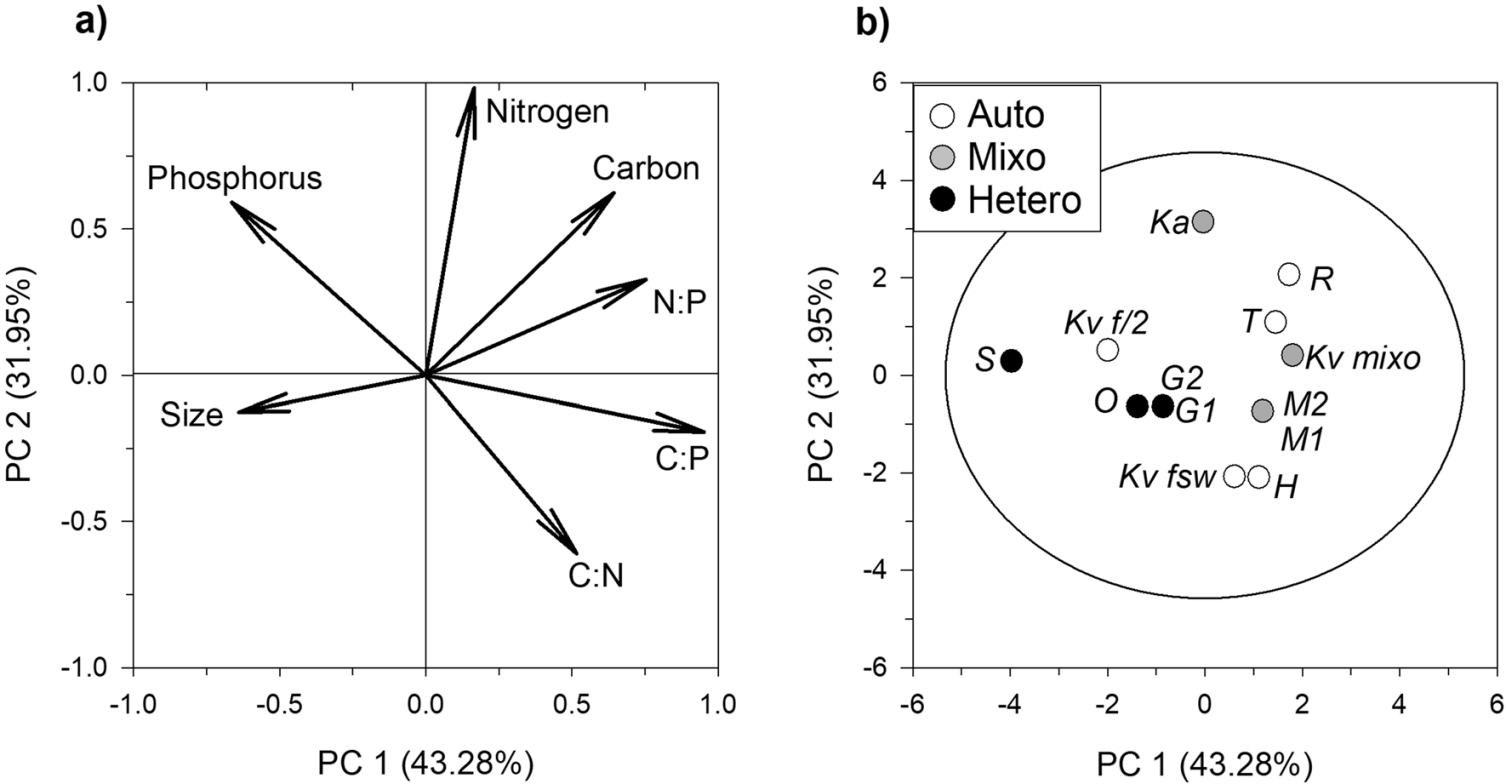
Principal component analysis. (**a**) Component loadings of the original variables and (**b**) component scores of combinations of the original variables along the two principal component axes. The circle in (**b**) depicts the Hotelling's T^2^ with 95% confidence interval. Abbreviations in (**b**) are described in Table 3.

Size, elemental contents and stoichiometric molar ratios of the prey species are listed in Table 1. The C content normalized to cell volume was lower in heterotrophs (0.11 ± 0.004 pg C μm^−3^) compared to the other trophic modes (0.15 ± 0.01 and 0.17 ± 0.02 pg C μm^−3^ in autotrophs and mixotrophs, respectively; Kruskal–Wallis, Dunn’s test, *P* = 0.041). N and P contents and stoichiometric ratios (C:N, C:P and N:P), however, did not differ among trophic modes (one-way ANOVA, *P* > 0.05). We further explored potential differences between the prey trophic modes by Principal Component Analysis (PCA). Figure 1 shows the contribution of prey stoichio-morphometric traits on the grouping of species within an orthogonal 2D-space along the two most relevant principal components (PCs). C:P ratio and N content explained > 90% of variation along the PC1 and PC2 respectively (Fig. 1a). However, no particular distinction among trophic modes emerged as auto-, mixo- and heterotrophic prey were clustered together (95% CI) without significant differences (*P* > 0.05) (Fig. 1b).

**Table 1:** Size (equivalent spherical diameter, ESD), size class, elemental content and stoichiometric molar ratios of the prey species. The prey carbon and cell concentrations used in the copepod feeding incubations are also provided. Average ± 1 s.e.m.

Diet	ESD (µm)	Size class	pg C µm^−3^	pg N µm^−3^	pg P µm^−3^	C:N	C:P	N:P	µg C L^−1^	cells mL^−1^
*Rhodomonas salina*	7.2	Small	0.17 ± 0.01	0.04 ± 0.003	0.004 ± 0.00001	5.3 ± 0.05	98 ± 8.02	18.6 ± 1.343	945 ± 4.4	29,964 ± 141
*Karlodinium veneficum f/2*	10.5	Small	0.13 ± 0.008	0.03 ± 0.002	0.006 ± 0.00002	5.7 ± 0.20	58 ± 3.86	10.2 ± 0.984	546 ± 3.7	6741 ± 46
*Karlodinium veneficum FSW*	11.5	Small	0.12 ± 0.007	0.02 ± 0.001	0.003 ± 0.00002	7.3 ± 0.03	97 ± 5.80	13.2 ± 0.776	498 ± 1.9	4821 ± 19
*Thalassiosira weissflogii*	13.5	Medium	0.17 ± 0.004	0.03 ± 0.001	0.004 ± 0.00002	6.0 ± 0.02	105 ± 2.602	17.4 ± 0.396	711 ± 4.9	3053 ± 21
*Heterocapsa sp.*	13.7	Medium	0.15 ± 0.005	0.02 ± 0.001	0.004 ± 0.00002	9.0 ± 0.05	110 ± 3.775	12.2 ± 0.383	539 ± 2.1	2490 ± 10
*Karlodinium veneficum mixo*	12.0	Small	0.18 ± 0.01	0.03 ± 0.001	0.004 ± 0.00003	7.1 ± 0.05	109 ± 5.751	15.4 ± 0.727	690 ± 8.3	4270 ± 12
*Karlodinium armiger*	16.1	Medium	0.18 ± 0.005	0.04 ± 0.002	0.006 ± 0.00005	5.1 ± 0.07	84 ± 2.48	16.5 ± 0.667	410 ± 1.6	845 ± 3
*Mesodinium rubrum*	18.9	Large	0.13 ± 0.006	0.03 ± 0.001	0.003 ± 0.00009	6.1 ± 0.10	113 ± 5.874	18.4 ± 0.865	616 ± 5.7	1297 ± 29
*Oxyrrhis marina*	16.4	Medium	0.10 ± 0.001	0.02 ± 0.000	0.004 ± 0.00006	5.1 ± 0.03	71 ± 1.47	13.9 ± 0.242	506 ± 3.7	2202 ± 19
*Gyrodinium dominans*	18.9	Large	0.12 ± 0.003	0.02 ± 0.002	0.004 ± 0.0003	5.7 ± 0.19	80 ± 6.55	14.1 ± 1.388	686 ± 6.0	1514 ± 18
*Strombidium arenicola*	30.7	Large	0.11 ± 0.006	0.03 ± 0.003	0.006 ± 0.0003	4.8 ± 0.24	49 ± 3.47	10.3 ± 1.104	300 ± 25.0	179 ± 15

Regarding the variation within *Karlodinium veneficum* grown in different conditions, the C content of *K. veneficum* increased significantly when acting as mixotroph (0.18 ± 0.01 pg C μm^−3^), whereas the cell quota was similar under high (0.13 ± 0.08 pg C μm^−3^) and low (0.12 ± 0.007 pg C μm^−3^) nutrient load in both autotrophic cultures (one-way ANOVA, Tukey HSD, *P* = 0.008; Table 1). The P content was statistically different among the three functionally growing *K. veneficum* cultures (one-way ANOVA, Tukey HSD, *P* < 0.001), being two-fold lower in the poor-nutrient autotrophic dinoflagellate with respect to the nutrient-replete one (respectively, 0.003 ± 0.00002 pg P μm^−3^ and 0.006 ± 0.0002 pg  P μm^−3^; Table 1). In the *K. veneficum* reared as functional autotroph in high-nutrients, C:N and C:P molar ratios were significantly lower than the nutrient-depleted *K. veneficum*, regardless of their nutritional strategies (one-way ANOVA, Tukey HSD, *P* < 0.001). However, the N:P ratio increased significantly in the mixotrophic *K. veneficum* compared to the nutrient-rich autotrophic dinoflagellate (Kruskal–Wallis, Tukey HSD, *P* = 0.03; Table 1).

### Ingestion rates

**Figure 2:**
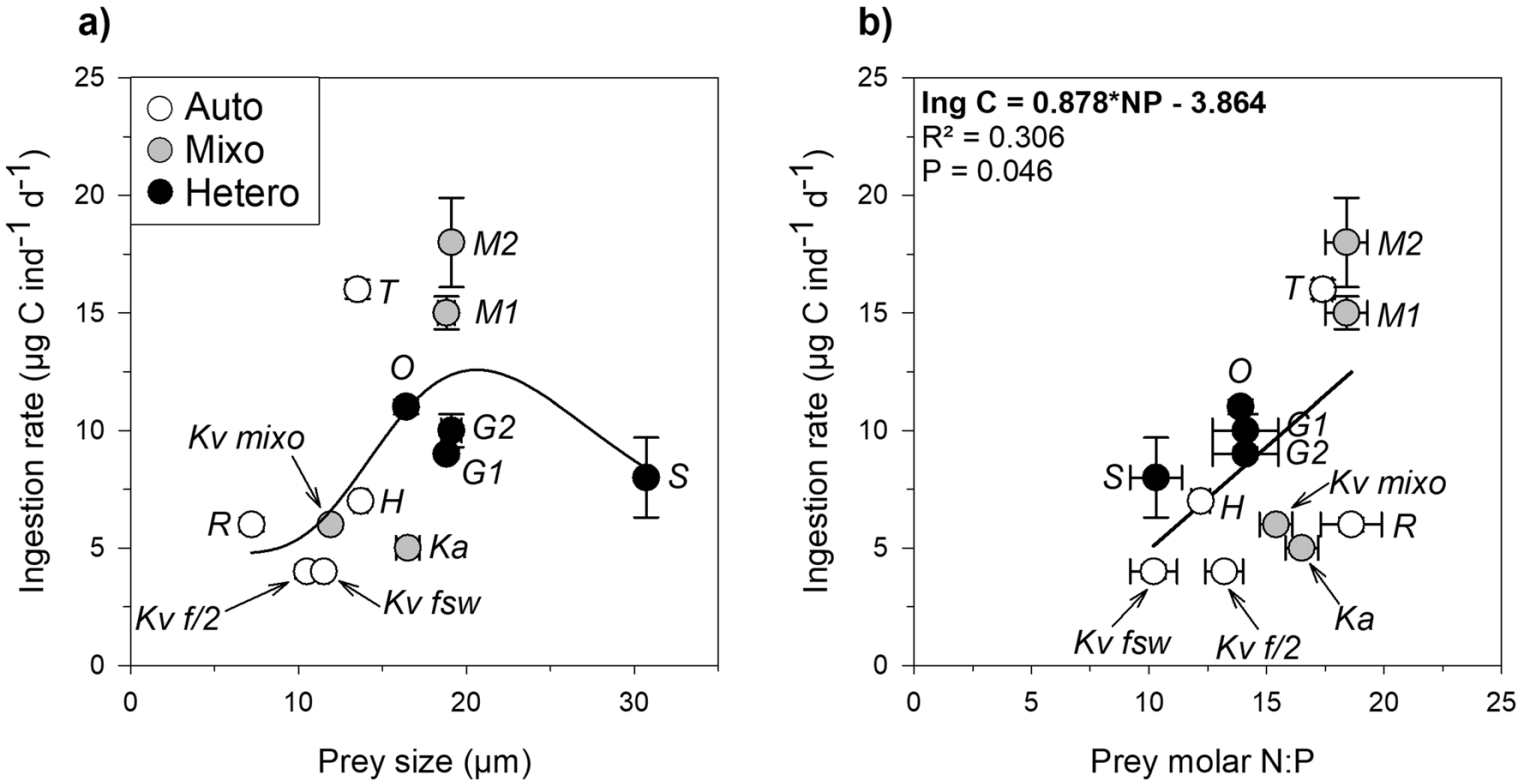
Ingestion rate of adult female *P. grani* as a function of (**a**) prey size and (**b**) prey N:P ratio. Non-linear fit with peak Sigma-Plot function in (**a**) and simple linear regression fit and corresponding equation in (**b**) are provided. Abbreviations are described in Table 3. Error bars are ± 1 s.e.m.

Figure 2a shows the ingestion rates of *P. grani* adult females, in terms of carbon intake, as a function of prey size. In general, there were significant differences in the feeding rates between size classes (7–13 µm; 13–18 µm; 18–31 µm) (one-way ANOVA, Bonferroni, *P* = 0.026). Mean values ranged between 3.7 ± 0.1 µg C ind^−1^ d^−1^ when fed the small-sized autotrophic *K. veneficum* (grown in FSW) and 18 ± 2 µg C ind^−1^ d^−1^ when fed the large-sized mixotrophic *Mesodinium rubrum*. Nevertheless, despite the large size, the aloricate ciliate *Strombidium arenicola* was eaten at moderately lower rates compared to the other large prey species (Fig. 2a). Even within the intermediate prey size range, substantial variation (ca. 3.5-fold change) was found among species. The ingestion rates did not vary significantly among functional trophic modes (one-way ANOVA, *P* = 0.547), most likely because of the high variability observed, especially among the different autotrophic and mixotrophic prey. Regarding stoichiometric ratios, we only found a moderate but significant linear relationship between C ingestion and prey N:P ratio (one-way ANOVA, *P* = 0.046, Fig. 2b; Table S1). Regarding *K. veneficum*, which was tested under the 3 different trophic conditions (Fig. 2a, Table 2), significant differences emerged between autotrophic and mixotrophic nutrition (Kruskal–Wallis, Tukey HSD, *P* = 0.038). In fact, the C ingestion of the adult female *P. grani* on the mixotrophic *K. veneficum* (5.9 ± 0.1 µg C ind^−1^ d^−1^) was ca. 1.6-fold higher than the one measured on the dinoflagellates grown autotrophically in f/2 (3.8 ± 0.3 µg C ind^−1^ d^−1^) and nutrient-poor (i.e. FSW) medium (3.7 ± 0.1 µg C ind^−1^ d^−1^).

**Table 2:** Copepod physiological responses under the three *K. veneficum* diets.

Physiological response	*K. veneficum* treatments		
	Auto (f/2)	Auto (FSW)	Mixo (FSW + *R. salina*)
Ingestion rate (µg C ind^−1^ d^−1^)	3.8 ± 0.32	3.7 ± 0.14	5.9 ± 0.14*
Fecal pellet production rate (fecal pellets ind^−1^ d^−1^)	12 ± 0.5	5 ± 0.2**	8 ± 0.3
Fecal pellet volume (10^6^ µm^3^ fecal pellet^−1^)	0.14 ± 0.013	0.16 ± 0.032	0.34 ± 0.042**
Egestion rate (10^6^ µm^3^ ind^−1^ d^−1^)	1.7 ± 0.07	0.9 ± 0.05*	2.8 ± 0.12**
E/I ratio (%)	5.8 ± 0.23	2.8 ± 0.15	8.4 ± 0.44*
Egg production rate (eggs ind^−1^ d^−1^)	27 ± 1.8	30 ± 2.2	25 ± 1.1
Gross-growth efficiency (%)	23 ± 2.5	29 ± 2.9	15 ± 0.5*
Hatching success (%)	71 ± 2.5	74 ± 2.0	72 ± 2.7

*Auto* autotrophic, *Mixo* mixotrophic, *FSW* plain filtered seawater.

Significant comparisons are indicated by *. *P < 0.05, **P < 0.001. Average ± 1 s.e.m.

### Fecal pellet production rates

**Figure 3:**
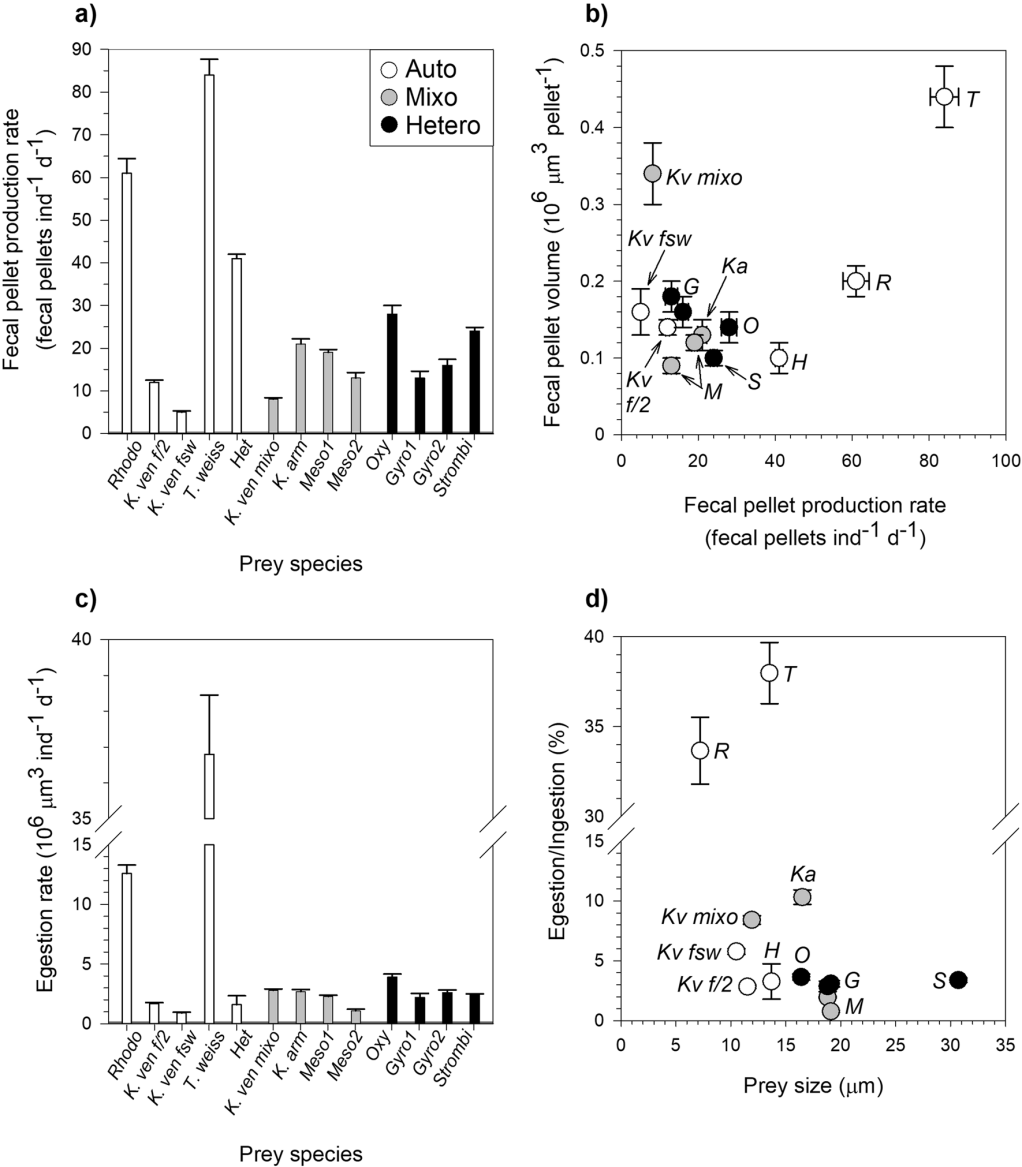
Egestion of *P. grani*. (**a**) Fecal pellet production rate, (**b**) fecal pellet volume as function of fecal pellet production rate, (**c**) volumetric egestion rate in the different dietary treatments, (**d**) egestion/ingestion ratio as function of prey size. Abbreviations are described in Table 3. Error bars are ± 1 s.e.m.

Fecal pellet production rate ranged between 5 ± 0.3 pellets ind^−1^ d^−1^ on a diet of *K. veneficum* (FSW) and 84 ± 4 pellets ind^−1^ d^−1^ when fed on *Thalassiosira weissflogii* (Fig. 3a). Fecal pellet size was variable and ranged between (geometric) mean values of 0.09 × 10^6^ µm^3^ pellet^−1^ for *M. rubrum* and 0.44 × 10^6^ µm^3^ pellet^−1^ for the diatom *T. weissflogii* (Fig. 3b). When egestion rate is expressed as total biovolume of fecal pellet produced, most of the prey tested rendered rather similar and low egestion rates (with values ranging between 0.9 ± 0.1 and 3.9 ± 0.3 × 10^6^ µm^3^ ind^−1^ d^−1^ for *K. veneficum* (FSW) and *Oxyrrhis marina,* respectively, Fig. 3c), with the exception of the cryptophyte *Rhodomonas salina* and the diatom *T. weissflogii*. On these two diets, in fact, the egestion rate of adult female *P. grani* accounted for 12.6 ± 0.7 × 10^6^ µm^3^ ind^−1^ d^−1^ (*R. salina*) and 37 ± 2 × 10^6^ µm^3^ ind^−1^ d^−1^ (*T. weissflogii*). These egestion rates were ca. 5 and 17 times higher than the average rates shown on the other prey. When comparing *K. veneficum* diets, as a consequence of increased pellet size, the mixotrophic *K. veneficum* yielded significantly higher egestion outputs compared to the autotrophic ones (one-way ANOVA, Tukey HSD, *P* < 0.001, Fig. 3b,c, Table 2). No correlation was found between defecation traits (pellet volume, pellet production and egestion rate) and either prey size or ingestion rate (data not shown; one-way ANOVA, *P* > 0.05 in all cases), nor with prey stoichiometry (one-way ANOVA, *P* > 0.05, Table S1).

The ratio of egested biovolume to total ingested biovolume (E/I ratio; Fig. 3d), expressed as a percentage, is as a proxy for discerning potential differences in either the absorption efficiency, the degree of pellet packaging or the presence of copepod sloppy feeding under the different diets. With the exception of *T. weissflogii* and *R. salina*, with E/I ratios of respectively 38 ± 2% and 34 ± 2%, for the rest of species the mean value was 4.2 ± 0.8% and the range of variation was moderate (1–10%; Fig. 3d), without significant differences across trophic modes or prey size classes (one-way ANOVA, *P* > 0.05) nor prey stoichiometry (one-way ANOVA, *P* > 0.05, Table S1). At a finer scale, intraspecific variations in E/I ratio emerged when the different *K. veneficum* diets were compared. In fact, a higher E/I ratio was observed under mixotrophic *K. veneficum* diet (8.4 ± 0.3%) compared to the autotrophic *K. veneficum* diets, either in high or low nutrient (FSW) media (respectively, 5.8 ± 0.2% and 2.8 ± 0.1%; Kruskal–Wallis, Tukey HSD, *P* = 0.020, Fig. 3d, Table 2).

### Egg production rates, egg hatching success and gross-growth efficiency

**Figure 4:**
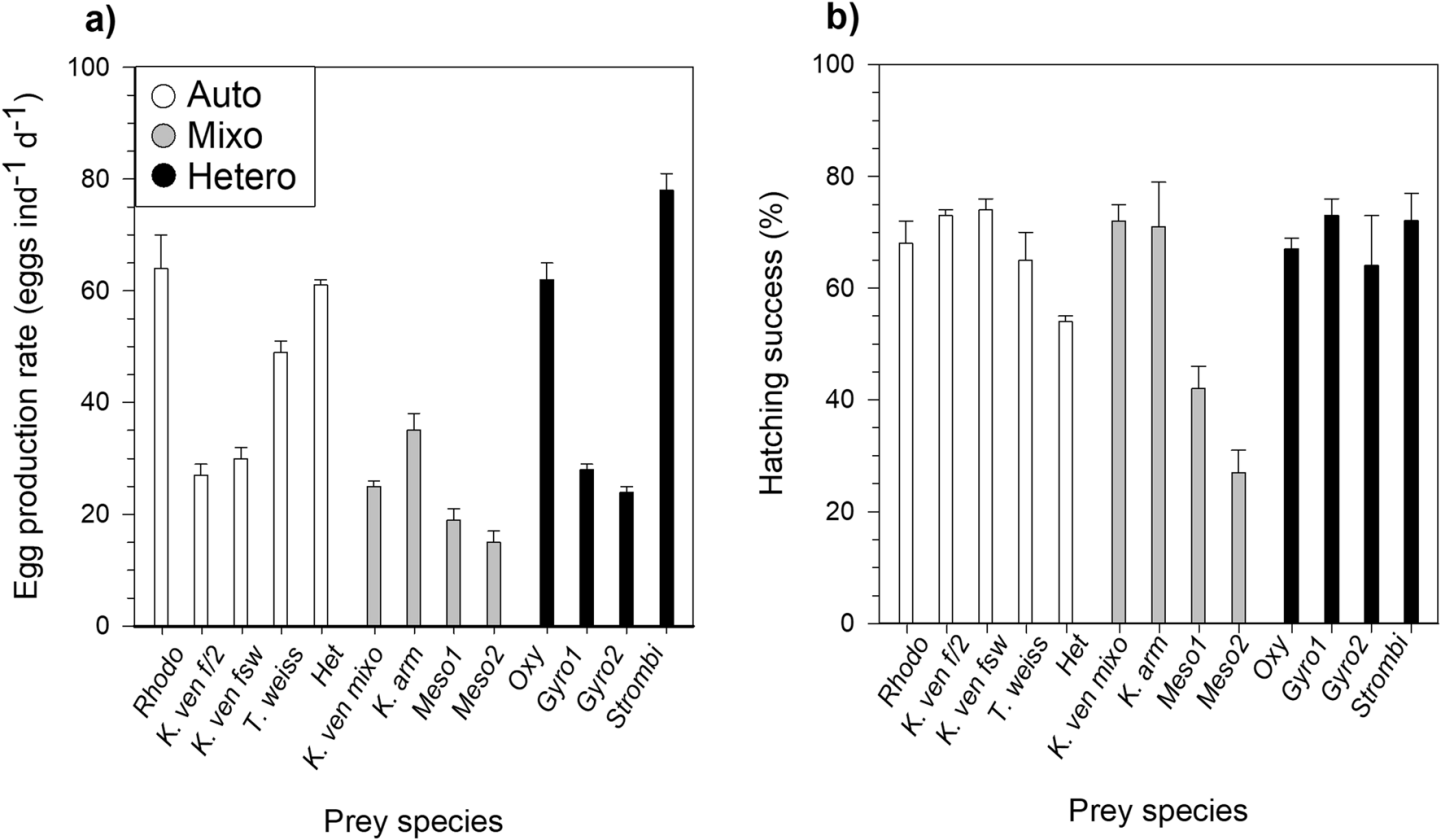
Reproduction and recruitment in the different dietary treatments. (**a**) *P. grani* egg production rate and (**b**) egg hatching success after 48 h from collection. Error bars are ± 1 s.e.m.

*P. grani* egg production rates were variable and ranged between 15 ± 2 eggs ind^−1^ d^−1^ when the diet consisted of *M. rubrum* and 78 ± 3 eggs ind^−1^ d^−1^ when fed the heterotrophic ciliate *S. arenicola* (Fig. 4a). The three *K. veneficum* cultures showed similar reproductive outputs (mean: 27 ± 1 eggs ind^−1^ d^−1^, Table 2). In either case, no significant difference in egg production rates among diets was found when comparing among trophic modes or between the three *K. veneficum* diets (one-way ANOVA, *P* > 0.05). Neither food quality (prey stoichiometric ratios) significantly influence egg production rate (one-way ANOVA, *P* > 0.05, Table S1).

Egg hatching success was overall high under most of the dietary treatments (nearly 70%; Fig. 4b), but declined when copepods were fed *M. rubrum* (27% ± 4) and to less extent when fed the autotrophic *Heterocapsa* sp. (54% ± 1). The three differentially growing *K. veneficum* did not exhibit any significant difference in hatching success among them (one-way ANOVA, *P* > 0.05; Fig. 4b, Table 2). Slightly significant relationship was found between hatching success and prey C:P ratio (one-way ANOVA, *P* = 0.042, Table S1). However, the amount of variance explained was rather low and the relationship was no longer significant when one outlier species (*M. rubrum*) was removed.

**Figure 5:**
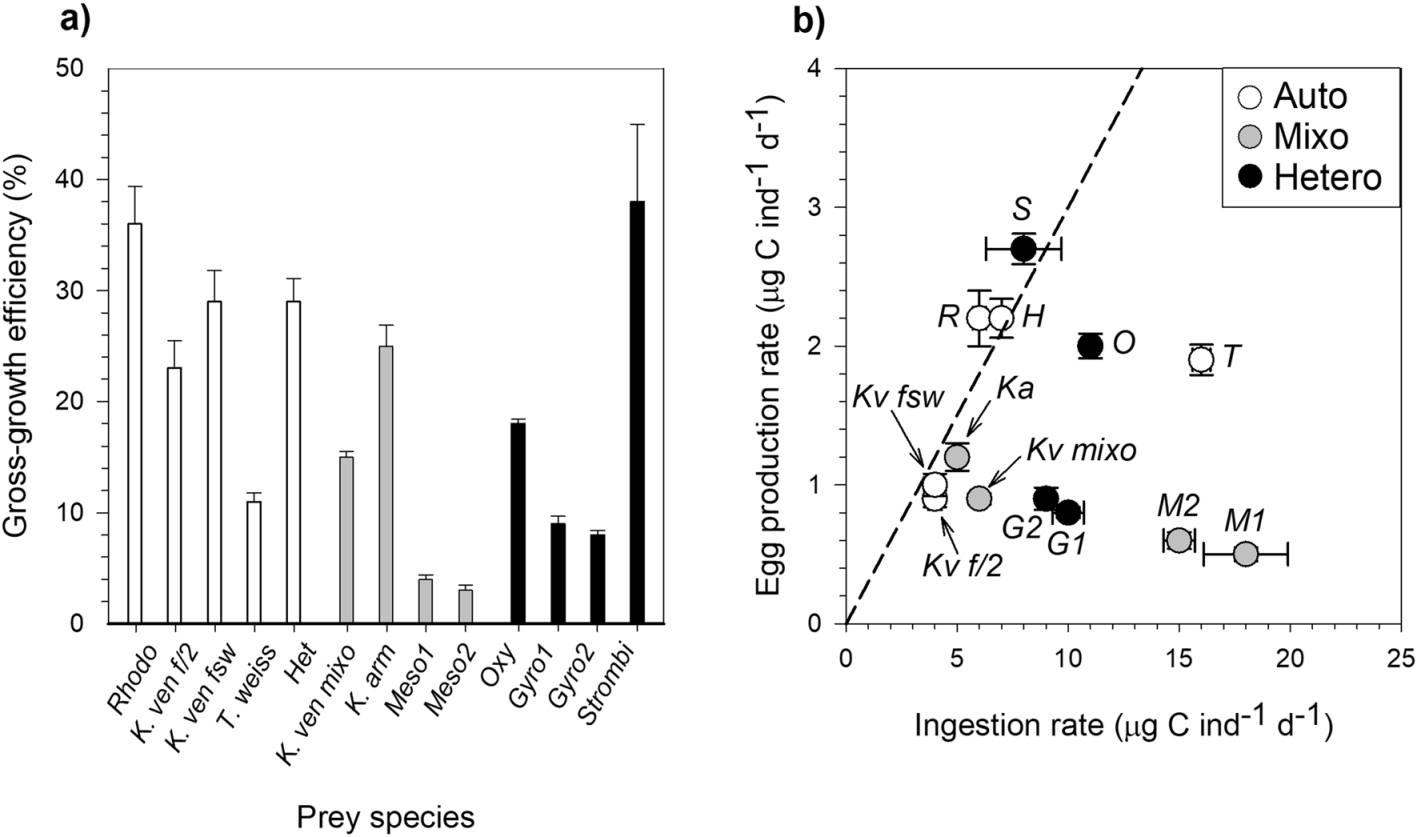
Gross-growth efficiency of *P. grani*. (**a**) Carbon gross-growth efficiency in the different dietary conditions and (**b**) egg production rate as a function of ingestion rate. The dashed line in (**b**) represents a 30% gross-growth efficiency illustrated for comparative purposes. Abbreviations are described in Table 3. Error bars are ± 1 s.e.m.

*P. grani* females showed high values (25–40%) of gross-growth efficiency when feeding on *R. salina*, *K. veneficum* (FSW), *Heterocapsa* sp., *Karlodinium armiger* and *S. arenicola* (Fig. 5a). Very low efficiencies (< 10%) were found when fed *M. rubrum* and *Gyrodinium dominans*. Surprisingly, the latter two prey were ingested at reasonably high rates, but the reproductive output was comparatively very low (Fig. 5b). No significant differences were found in gross-growth efficiencies of *P. grani* on the different diets according to the nutritional mode of the offered prey (one-way ANOVA, *P* > 0.05; Fig. 5a) and no significant relationship existed with prey food quality (one-way ANOVA, *P* > 0.05, Table S1). Contrarily to this general picture, however, when we focused only on *K. veneficum,* the consumption of the autotrophic and mixotrophic cultures yielded different gross-growth efficiency outputs. In fact, the mixotrophic diet resulted in lower *P. grani* gross-growth efficiency (15% ± 1) compared to autotrophic nutrition in either high or low nutrient conditions (respectively, 23% ± 2 and 29% ± 3), without differences between nutrient treatments (Kruskal–Wallis, Tukey HSD, *P* = 0.03; Fig. 5a, Table 2).

## Discussion

Phagotrophic organisms are expected to accumulate more N and P compared to autotrophs that store C-rich structural polysaccharides and photosynthates^27^. However, we found no significant differences in the elemental contents and stoichiometric ratios among the different nutrition modes of protist prey (Fig. 1, Table 1). Only the C content of the heterotrophs was significantly lower than that for the autotrophic and mixotrophic prey, but this difference did not translate into significant stoichiometric changes (Table 1). The lack of clear differences in the stoichiometric ratios observed when comparing our autotrophic and mixotrophic species (and strains) may be due to (a) stronger but species-specific dependency of autotrophs on external inorganics and (b) flexible reliance of mixoplankton on either nutritional mode under the applied growing conditions. In fact, mixoplankton can lie in the phototrophic or heterotrophic spectrum of the trophic continuum according to the mixotype^16^. Furthermore, beside the well-known role of nutrients and light, also the interplay between these two and the size class is acknowledged to influence stoichiometry in protists within a given trophic category^27^. Among the species tested, cell volume-normalised C content (including N and P also when reported) was coherent with previously-reported values in the literature for the autotrophs *R. salina* and *T. weissflogii*^28^, *Heterocapsa* sp.^29^ and the heterotrophs *G. dominans* and *O. marina*^30^, and the closely-related *Strombidium sulcatum*^31^ under similar growth conditions than those in this study. In the kleptoplastidic *M. rubrum*, elemental quotas normalised to cell volume were slightly different from those of a previous study^32^, perhaps due to the differences between works in the nutrient load of the growth medium. We could not find any elemental content profile in the literature for *K. armiger*, but our C estimate agreed well with the ones calculated on the basis of general equations developed for athecate dinoflagellates^33^.

Regarding the elemental composition of our autotrophic *K. veneficum* strain, our values matched those reported by Li et al. under Redfield nutrient proportions^34^. Some authors have reported a correlation between the ingestion rate of *K. veneficum* and the predator C quota^34^, which might explain the increase in C content observed in our mixotrophic *K. veneficum* compared to the phototrophic cultures (Table 1). Generally, small-intermediate sized mixoplankton tend to feed on C-rich phototrophs when light or nitrogen-depleted^27^. Furthermore, it is worth noting that karlotoxins are C-rich structures, which are produced in higher proportion under nutrient limitation and during active feeding^35,36^. Then, it is possible that the C quota of the mixotrophic *K. veneficum* was higher than that of autotrophic cultures due to the use of karlotoxins for phagotrophy^34^. We unfortunately did not measure toxin content in our experiments.

The ingestion rates of *P. grani* female fell within the range of values previously reported for this copepod species under high concentrations of *R. salina*^37^, *Heterocapsa* sp.^38^, *T. weissflogii*^39^ and *O. marina*^40^, and for the closely-related *Acartia tonsa* on similarly-sized prey^39,41,42^. Regarding stoichiometric ratios as proxy of food quality, we found a relationship between copepod ingestion rate and prey N:P ratio (Fig. 2b), indicating that P-poor prey were ingested at higher rate, probably as a means to compensate for the lack of the specific nutrient^43^, although copepod response to nutrient deficient-prey may also be unaffected as previously demonstrated^38^. However, the amount of variance in ingestion rates explained by prey N:P ratio was rather modest (30%), and was basically driven by the data relative to *M. rubrum* and *T. weissflogii*. Conversely, as could be anticipated, prey size appeared to be the most relevant factor to consider when interpreting our results. Thus, ingestion rates of *P. grani* increased with prey size up to an optimum, and then declined when fed the largest prey tested, *S. arenicola*, resulting in values similar to those exhibited for much smaller prey species (i.e., *Heterocapsa* sp.) (Fig. 2a). Such dome-shaped relationship between feeding rates and prey size are already known for copepods^3,28,44^. Very small prey are hardly perceived or retained efficiently, whereas larger prey may either exhibit strong escape responses or require longer handling by the copepod^45^ or being too big to be fully ingested^3^. The optimal prey size range (13–18 µm) for *P. grani* observed in our study fits well with the values reported for adult *A. tonsa*^44^.

Although egestion rates and pellet size of *P. grani* varied largely among prey, they were not correlated with either ingestion rates or prey size and were independent of prey stoichiometry (Table S1) or of prey nutritional strategy (Fig. 3a–c), as also reported for *A. tonsa* fed different autotrophic and heterotrophic protists^42,46^. Copepod fecal pellet size and density have been shown to be either related to^47^ or independent of food concentration. However, in our experiments, copepods were always above satiation level, thus the differences reported herein must result from species-specific particularities in the degree of prey packaging. Our pellet volume estimates agree with previous reports on *A. tonsa* fed saturating concentration of various prey over a size range between 7 and 20 µm^42^. The large pellet volume on a diatom-diet was expected, since diatom siliceous materials are not digested within copepod intestinal tract and fecal pellets appear larger and more resistant compared to the ones egested under soft diet^42,48,49^. The increase in pellet size recorded in the mixotrophic *K. veneficum* diet, compared to the two autotrophic cultures of this species, may be the consequence of the phagotrophic activity of the dinoflagellate on *R. salina*. It is possible that the dinoflagellate might change composition when grown mixotrophically^50^, yielding different pellet volume in copepods. In general terms, enhanced egestion rates due to increase in pellet dimension under functionally mixotrophic *K. veneficum* diet (Table 2) would have an influence in the recycling of organic matter within the microbial food web, as fecal pellet size (and density) normally determines their sinking rates, modifying the export production out of the photic layer^42^.

The analysis of the egestion/ingestion (E/I) ratio, based on volumetric data (Fig. 3d), may be more informative than the sole comparison of egestion rates, as it considers the amount (volume) of prey ingested. The calculated E/I ratios overall had no clear relationship with prey size, prey stoichiometry or prey trophic mode, showing distinctively high values when copepods were fed the cryptophyte *R. salina* and the diatom *T. weissflogii*. Comparing our E/I ratio for *T. weissflogii* (44%) with those obtained by Besiktepe and Dam (58%)^42^, it is rather clear that the diatom is largely undigested by *Acartia* spp*.* due to the presence of a siliceous frustule. In contrast, our E/I ratio for *R. salina* (33%) is higher than those obtained in their study for similarly-sized soft prey such as *Dunaliella tertiolecta* (4%) and *Uronema* sp. (7%)^42^*.* The reason for the high E/I ratio when fed *R. salina* is less clear. Assuming the copepod absorption efficiency to be the reciprocal of the E/I ratio, our estimated copepod absorption efficiency (on a volumetric basis) would be 63% for *R. salina* and 62% for *T. weissflogii*, whereas in all the other prey tested the values fell within the very high range (> 90%). In this regard, Thor and Wendt^51^ compared the carbon absorption efficiency of *A. tonsa* fed different prey^51^. These authors found that at high *R. salina* concentrations the copepod carbon absorption efficiency was particularly lower (53%) than when fed other species (*T. weiisflogii*: 63%, *D. terctiolecta*: 77%), hence resulting in much higher expected egestion rates, in agreement with our results^51^.

Ingested food is eventually respired, egested and excreted as discarded materials and solutes, and when exceeding the basal metabolic requirements, it is finally incorporated into the body as reserves, growth or reproductive output. In our experiments*, P. grani* egg production rate increased with ingestion rate, maximum values reached on a diet of the heterotrophic ciliate *S. arenicola*, closely followed by *R. salina*, *O. marina* and *Heterocapsa* sp. (Fig. 4a). The egg production rates obtained in our experiments match previous values reported on *P. grani* feeding on some of the tested prey (*O. marina*, *R. salina*, *Heterocapsa sp*.)^38,40,52^. However, the increase in egg production was not always coherent with ingestion rate. In fact, surprisingly, *T. weissflogii*, *M. rubrum* and *G. dominans*, which fall in the preferred prey size range (13–18 µm), yielded rather low reproductive output compared to the corresponding ingestion rate (Fig. 5b). In the case of the diatom, the presence of silica frustule^48^ and the abovementioned drop in absorption efficiency may help explain the lower fecundity. Since no relationship was found between egg production and prey stoichiometric traits (Table S1), it is possible that the prey which resulted in comparatively low egg production rates were presumably deficient in some essential fatty acids or other compounds essential to copepod reproduction and recruitment^8^. Unexpectedly, the egg production rate of *P. grani* when offered *K. veneficum* was similar between mixotrophic and autotrophic diets, regardless of the nutrient conditions (Table 2). We cannot discard, however, that the lack of apparent advantage in the reproductive output for copepods fed mixoplankton could be related to the degree of mixotrophic behaviour exhibited by our *K. veneficum*, with rather moderate ingestion rates (maximum daily intakes of 1 *R. salina* grazer^−1^^53^). *K. armiger*, on the other hand, promoted higher egg production rate. *K. armiger* is known to graze on a greater extent (ca. 9 *R. salina* grazer^−1^ d^−1^) compared to the congeneric *K. veneficum*^54^, thus, perhaps contributing to enhanced nutrient transfer to the predator in our study.

Our main aim was to assess the gross-growth efficiency of copepods when fed diets of different nutritional mode. Although egg production rates per se are a very useful proxy to understand the overall impact of a prey on secondary production, the normalization of the reproductive rate per amount of food ingested allows a better picture of the prey nutritional quality. The gross-growth efficiency for C in marine calanoid copepods under optimal conditions usually falls around 30–40%^44,55^. Without distinction on the basis of prey trophic mode, several of the diets we tested reached these high efficiency values in agreement with other studies^56,57^. Other diets, however, proved to be less efficient, e.g., *M. rubrum* and *G. dominans* (Fig. 5a). Considering the dependency of ingestion on prey size, one may argue that prey size constraints (i.e. sloppy feeding) could be the source of the particularly low gross-growth efficiency (3–8%) measured for these large species. Large prey might not be ingested in their entirety, therefore resulting in overestimation of actual ingestion rates when the food removal methods is applied^3,4^. Using the equations provided by Møller^4^ to calculate the expected prey size-dependent loss of organic matter by copepod sloppy feeding, the overestimation of C intake due to sloppy feeding in our study would account for up to 13%. Such augmented values would negligibly affect our gross-growth efficiency estimates (only 0.6 and 1.4% increase for the *M. rubrum* and *G. dominans* diets, respectively). Therefore, these corrections assuming sloppy feeding would not be sufficient to explain the huge discrepancy found between ingestion and reproduction under these diets. Moreover, we did not observe lower gross-growth efficiency in *P. grani* when fed even larger prey, *S. arenicola*, confirming that sloppy feeding alone cannot be the source of low reproductive output under *M. rubrum* and *G. dominans* diets. Alternatively, we may suggest toxicity (as observed in some diatom species^58^) or deficient food quality in these prey^7,30,56^. Concerning low nutritional quality arguments (stoichiometric constraints), one would expect some compensatory feeding as demonstrated to occur in *A. tonsa* fed nutrient-deficient algae^43^. In our study, high N:P prey such as *M. rubrum* were ingested at higher rates (Fig. 2b). Yet, no significant relationship emerged between gross-growth efficiency and prey N:P ratio (Table S1), hence rendering stoichiometric arguments rather weak in explaining the variations observed. Additionally, as mentioned before, fatty acids are essential dietary constituents in copepod reproduction. We did not determine the fatty acid contents and composition of our tested prey, but for some of them fatty acid data can be found in the literature and may help to interpret our results. Thus, in accordance with our results for *M. rubrum* and *G. dominans*, Broglio et al*.*^7^ found that diets of the heterotrophic *M. pulex* and *G. dominans* were less nutritional to *A. tonsa* compared to the cryptophyte *R. salina*. A positive correlation was found between reproduction and ingested fatty acids (especially the ratio between 22:6ω3 and 20:5ω3), but no specific dependency was determined on the basis of prey nutritional strategy, reinforcing our observations^7^. In their study the low performance of *M. pulex* and *G. dominans* was attributed to the lower 22:20 ratio, which is known to influence the reproductive performance in crustaceans^7^.

Concerning the effects of trophic mode on the nutritional value of *K. veneficum*, we observed a drop in gross-growth efficiency when copepods were fed on the dinoflagellate acting as mixoplankton, contrarily to literature and model expectations (Fig. 5a, Table 2). As mentioned before, *K. veneficum* exhibits variation in the phagotrophic tendency^22,53^ and produces karlotoxins (although with a large variability between strains^35^) to immobilize its prey prior to capture. Therefore, it could be hypothesized a possible involvement of toxic weapons against *R. salina* on which the dinoflagellate was feeding before being offered to the copepods^36^. Nevertheless, in our experiments any possible allelopathic effects were not enough to trigger deadly outcomes on the copepods throughout the 5 days of exposure and, although the gross-growth efficiency of the copepod was clearly lower, this was still comparable to those yielded under other non-toxic diets (*O. marina*, *Heterocapsa* sp., *T. weissflogii*). Although *K. veneficum* has been reported toxic in occasions for copepods, in our experiments we did not observe any enhanced mortality or sublethal effects (abnormal motility) compared to the other prey. Furthermore, in other independent experiments (unpublished data), we observed that the mortality of *P. grani* along ontogeny on a diet of both functionally autotrophic (20%) and mixotrophic *K. veneficum* (16%) was overall comparable to that registered on a monodiet of *R. salina* (20%) (Traboni et al., unpubl). This renders *K. veneficum* suitable prey for *P. grani* and valid for comparison with the other species tested, despite its acknowledged toxicity. The gross-growth efficiency on a diet of *K. armiger* was considerably high, despite the species has been demonstrated to have detrimental acute effects on other *Acartia* species when offered at saturating concentrations^22^. Perhaps toxicity is boosted upon ammonium addition as nitrogen source^59^, which was not the case in our culture medium. This hypothesis stresses the importance of nutrient types other than ratios for mixotrophic growth and toxicity to be tested in future experiments.

Finally, egg hatching success was, in most cases, relatively high and differences recorded were not related to the prey trophic mode neither across diets nor among *K. veneficum* trophic types (Fig. 4b). For *Acartia* sp., similar or slightly higher hatching rates were reported on natural plankton assemblage as well as on monodiets of *R. salina, G. dominans* and *O. marina* in the range between 67 and 91%^40,45,52,60^. Our hatching rates dropped in only a few cases, specifically on a diet of *M. rubrum* and *Heterocapsa* sp. In Broglio et al.^7^, lower hatching (20–60%) was recorded in *A. tonsa* fed the ciliate *S. sulcatum* and more generally hatching was found to be related to the ingestion of certain polyunsaturated fatty acids (PUFAs), stressing the importance of fatty acid composition for egg viability^7^. The same hypothesis, however, cannot be made for *Heterocapsa* sp., whose reported content of cellular PUFA^57^ is similar to that of other species which yielded high hatching rates in the above mentioned studies. Phosphorus depletion has been proposed as additional limiting conditions for copepod development in both field^61^ and laboratory experiments^38^. Even if moderate, we observed increased hatching rate in copepods fed low C:P prey (Table S1); yet, this relationship seems to be no longer significant if high-C:P *M. rubrum* is removed. Although copepod C-ingestion and prey N:P ratio were positively related, the calculated P ingestion rates on *M. rubrum* and *Heterocapsa* sp. are not different from other species tested (between 1 and 4 × 10^–7^ µg P ind^−1^ d^−1^) and cannot explain the variation in hatching rates we observed.

To our knowledge this is the first attempt at evaluating the actual nutritional contribution that mixoplankton may have on marine copepods. Most studies that have focused on mixotrophic species as prey for copepod feeding experiments have not used functionally phagotrophic mixoplankton (i.e. feeding on other protists). Although until relatively recently mixoplankton was considered a rare fact, the current gap in the empirical observation of mixoplankton role in copepod diet is also a consequence of the challenge in reaching considerable standing stocks for experimentation under controlled phagotrophic status^15^.

Overall, we found that only moderate relationship existed between copepod ingestion rate with the prey N:P ratio and size. None of the reproductive parameters was substantially affected by nutritional quality proxies due to the intraspecific variability among prey traits and the lack of a clear pattern placing mixotrophs on a different nutritional quality level with respect to other protists, according to their stoichiometry. We conclude that in the conditions tested mixoplankton proved to be as nutritional as the strict phago- and autotrophs to *P. grani.* Mixotrophic *K. veneficum* diet under our conditions did not enhance copepod gross-growth efficiency in remarkable fashion compared to the obligate autotrophic dinoflagellates; instead, it represented a less valuable prey, perhaps due to a presumably certain degree of toxicity associated to the phagotrophic behaviour, and it promoted larger fecal pellets with potential consequences on C export rate. We postulate that our experimental conditions could not trigger significant variation in copepod physiology because of the resemblance of our mixotrophic strains to strict photo- or heterotrophic food. In other words, the limited reliance of *K. veneficum* on phagotrophy could have flattened important differences on copepod performance relative to strict autotrophic *K. veneficum* diets. Additional studies with other mixoplankters and other copepod species may help to provide clearer and more robust conclusions on the role of mixotrophy on the carbon transfer mediated by copepods to upper trophic levels. Moreover, since nutrient ratios are known to be environmental forcing triggering a switch in protist trophic mode, we acknowledge the need to assess in future research whether mixoplankton may change their quality and influence higher predators as a result of stronger nutrient regime shifts and higher prey availability.

## Materials and methods

### Copepod cultures

The calanoid copepod *Paracartia grani* used in the experiments originates from a culture maintained at the ICM^52^. The copepods were grown in 20–40 L tanks in a cold room at 19 °C and 10:14 light:dark cycle, and routinely fed the cryptophyte *Rhodomonas salina*. New copepod cohorts were set-up by siphoning the bottom of the culture tanks, and transferring the newly-collected eggs into a new tank. After hatching, the copepods were supplied with increasing concentrations of *R. salina* along development up to approx. 30,000 cells mL^−1^ (1000 µg C L^−1^) when adults. Only less than 2-week old healthy adult females were used in the experiments.

### Protist cultures

All prey species were grown non-axenically in batch cultures at 38 psu and 19 °C. The functionally autotrophic species tested were the cryptophyte *R. salina*, the diatom *Thalassiosira weissflogii* and the thecate dinoflagellate *Heterocapsa* sp., all growing exponentially in constantly aerated f/2 medium (+Si for *T. weissflogii*) under high light (90–100 μmol photons m^−2^ s^−1^). *Heterocapsa* and *Rhodomonas* species have been reported to be able to feed on bacteria when environmental conditions challenge the strict autotrophic metabolism (5–13 bacteria protist^−1^ h^−1^ in *Heterocapsa rotundata* by food removal method^62^ and < 1 bacteria protist^−1^ in *Rhodomonas sp*. analysing the food vacuole content^63^). However, in our conditions, we assume that plenty of inorganic nutrients and high irradiance levels would limit bacterial uptake to negligible rates, conferring both species in our study the role of autotrophs. As strict heterotrophic protists, cultures of the athecate dinoflagellates *Gyrodinium dominans* and *Oxyrrhis marina* and of the ciliate *Strombidium arenicola* were grown in non-aerated filtered seawater (FSW) with a daily supply of *R. salina*. Finally, we selected the non-constitutive mixoplanktonic ciliate *Mesodinium rubrum* (reared in non-aerated FSW on a diet of the cryptophyte *Teleaulax amphioxeia*) and two athecate dinoflagellates, *Karlodinium veneficum* and *Karlodinium armiger* as constitutive mixoplankton; both *Karlodinium* species were reared in FSW and supplied *R. salina* during the culturing period. In the case of *K. veneficum*, we further explored the effects of nutrient status and trophic strategy “within species”. Thus, *K. veneficum* was grown in constant aeration both as strict autotrophic under two contrasted nutrient environments (f/2 medium vs. FSW), and as mixotroph in low-nutrient conditions (FSW + *R. salina*). This species is more flexible than our *K. armiger* strain and *M. rubrum* as the latter depend on food supply for their growth^64,65^, limiting our possibilities of testing them as pure autotrophs. Generally, to prevent overgrowth of prey in the mixoplankton and heterotrophic cultures, grazers were kept in semi-saturating food conditions. Further details in growth conditions can be found in Table 3. Standing stocks of the cultures were determined with a Multisizer III particle counter (Beckman Coulter) or by Sedgewick-Rafter counts.

**Table 3:** Growth conditions of the different prey species.

FTM	Species	Strain	Culture medium	Irradiance (µE)	Diet	Prey:Predator ratio	Culture volume (L)
Auto	*Rhodomonas salina (R)*	K-0294	f/2	110	*–*	–	5
Auto	*Karlodinium veneficum f/2 (Kv f/2)*	K21-ICMB-274	f/2	90	*–*	–	4
Auto	*Karlodinium veneficum fsw (Kv fsw)*	K21-ICMB-274	FSW	90	–	–	4
Auto	*Thalassiosira weissflogii (T)*		f/2 + Si	90	–	–	1
Auto	*Heterocapsa sp. (H)*		f/2	90	–	–	1
Mixo	*Karlodinium veneficum mixo (Kv mixo)*	K21-ICMB-274	FSW	90	*R. salina*	0.3	4
Mixo	*Karlodinium armiger (Ka)*	ICM-ZOO-KA001	FSW	40	*R. salina*	6	10
Mixo	*Mesodinium rubrum 1 (M1, M2)*	DK-2009	FSW	80	*T. amphioxeia*	2	20
Hetero	*Oxyrrhis marina (O)*	ICM-ZOO-OM001	FSW	20	*R. salina*	15	1
Hetero	*Gyrodinium dominans 1 (G1, G2)*	ICM-ZOO-GD001	FSW	20	*R. salina*	20	3
Hetero	*Strombidium arenicola (S)*	ICM-ZOO-SA001	FSW	20	*R. salina*	100	8

*FTM* functional trophic mode, *Auto* autotrophic, *Mixo* mixotrophic, *Hetero* heterotrophic. Letters in parenthesis after the species name refer to the abbreviated initials used in graphical representations. *FSW* plain filtered seawater containing 8.8 µM N (NO_2_^−^ + NO_3_^−^) and 0.5 µM PO_4_^3−^; *µE* µmol photons m^−1^ s^-1^. Notice that two experiments were carried out with *M. rubrum* (M1 and M2) and also with *G. dominans* (G1 and G2).

### Experimental setting

Copepod experiments consisted of a preconditioning period (4–5 days) followed by 24 h incubations for the determination of ingestion and egg production rates; both the preconditioning and experimental periods were carried out at saturating food concentrations (Table 1). We chose the long preconditioning to minimize food history effects on egg production^66^. Protist cultures were up-scaled to different final volumes based on experimental needs (Table 3). Batch cultures of the autotrophic prey were diluted on a daily basis to keep them in exponential growth. Regarding the mixo- and heterotrophs, when sufficient amounts were reached, we split the standing stocks into 5 1-L Pyrex bottles (or round-bottom flasks). These 5 individual new stocks were served to feed the copepods during each of the 4 preconditioning days and the experimental day. All grazer cultures were routinely food-replenished except the ones to be immediately used, which were left 24 h in starvation before being offered to the copepods, allowing the depletion of their own prey. Only *S. arenicola* and *O. marina*, which exhibited very fast prey removal, were fed until the last 24 h to avoid potential nutritional impoverishment. By following this protocol, copepods ingested prey of the same food quality throughout the preconditioning and experimental periods. *R. salina* and *T. amphioxeia* concentrations in mixo- and heterotrophic protist cultures were determined with the particle counter and/or by microscopic counts, ensuring no substantial cryptophyte biomass would be available to copepods in food suspensions (< 0.1 µg C L^−1^). Prey suspensions for copepod experiments were prepared by diluting the culture stocks to the desired concentrations, and adjusted with the Multisizer particle counter, which served for both enumeration of prey suspensions and estimation of cell volume. In the case of the ciliates *M. rubrum* and *S. arenicola*, which may escape from the particle counter flow, 10 mL samples were preserved in acetic Lugol’s solution (2% final concentration), and their concentrations determined under the microscope. For these latter species, however, cell volume was measured from live counts with the particle counter.

For preconditioning, groups of adult females (60–200 according to experimental needs) were sorted from the cohorts and transferred into 4 L Nalgene polycarbonate bottles filled with the respective food suspensions prepared in FSW (Table 1). Each bottle was amended with 5 mL of f/2 medium L^−1^ (f/2 + Si for *T. weissflogii*) to minimize the effects of copepod excretion^31^. Bottles were maintained at low irradiance (10 μmol photons m^−2^ s^−1^) to avoid prey overgrowth. Every day, 75% of the content of each preconditioning bottle was siphoned out with a silicone tube fitted with 20–40 µm mesh net (according to prey size) to avoid losing the copepods, and subsequently the bottles were refilled with fresh prey suspensions at the corresponding concentrations (Table 1). By this way, we ensured minimal stress and no loss of animals during refilling procedures.

The day of the experiment, 613 mL wide-mouth Pyrex bottles were carefully filled with new food suspensions (Table 1) in four sequential steps, gently mixing before refilling. The 24 h incubations comprised one start (to account for the initial prey concentration), four control (copepod-free) and four grazing (with added copepods) bottles. Copepods were retrieved from the preconditioning bottles by gentle filtration on a 200 µm mesh sieve, transferred to a Petri dish and then pipetted out into the experimental Pyrex bottles. The number of copepods used (8–26), reflected the prey size and the a priori expected clearance rates^37,40^. Thereafter, the start bottle was sacrificed to assess the initial prey concentration, and the remaining bottles were capped, checked for the presence of air bubbles and placed on a rotating Ferris wheel (0.2 rpm). Some extra copepods were fixed in formaldehyde (4% final concentration) and photographed under the inverted microscope (40 × magnification) for size measurements. The prosome length was determined with the software ImageJ.

After 20–24 h, the bottles were removed from the wheel and their contents filtered through 200 µm sieves to collect the copepods; control bottles were treated similarly for consistency. Copepods were checked for their condition, anesthetized with MS222^52^ and counted. Then, the 200-µm sieved water was thoroughly homogenized and 50–100 mL samples were taken for either the particle counter or microscopic counts (Lugol’s solution samples). Finally, the whole remaining content (400–500 mL) was filtered onto a 20 μm sieve (rinsed three times) to collect eggs and fecal pellets. These were transferred onto Petri dishes where fecal pellets were counted fresh after collection to calculate egestion rate. After counting, fecal pellets and eggs were photographed (100–200 × magnification; LEICA-MC170 HD) and then promptly incubated at 19 °C. After 48 h, the Petri dishes containing eggs and fecal pellets were preserved with Lugol’s solution; unhatched eggs and nauplii were enumerated to calculate egg production and hatching success. Egg shells contained in fecal pellets were taken into account for egg production rates^39^. Eggs and fecal pellet size was measured using the software ImageJ. Contours of eggs/pellets were traced and fit to a geometrical ellipsoid for the posterior calculation of biovolume.

Copepod feeding rates were calculated from prey removal according to Frost’s equations^67^. Egestion rates were expressed as number of fecal pellets and also as biovolume of fecal pellet produced. The ratio of egestion rate to ingestion rate in biovolume terms, E/I ratio, was calculated as the quotient between egestion rates and ingestion rates (both in µm^3^ ind^−1^ d^−1^ units), expressed as percentage.

Hatching success was estimated as percentage of nauplii hatched after 48 h, based on the initial egg number. Ingestion and egg production rates were converted into µg C_prey_ ind^−1^ d^−1^ using respectively the prey carbon contents analysed in this study (see below) and the *P. grani* egg content provided by Saiz et al.^68^. Gross-growth efficiency (as %) was calculated as the quotient between egg production rates and ingestion rates, both expressed in carbon terms.

### Elemental analysis

Prior to each experimental incubation, we filtered aliquots of the prey stocks onto pre-combusted (450 °C, 5 h) GF/F filters (Whatman, 25 mm) for determination of the carbon (C), nitrogen (N) and phosphorus (P) elemental composition. The filters for CN analysis were oven-dried at 60 °C, 48–72 h and then stored in a desiccator until processing with a Flash EA1112 (Thermo Finnigan) CHNS analyser. The filters for P analysis were immediately frozen at −80 °C until processing. P samples were processed as in Isari et al*.*^38^ applying the acid persulfate digestion method and posterior conversion to dissolved inorganic P with a Seal Analytical AA3 (Bran + Luebbe) analyser. In a few occasions, CNP profiles of the prey were not concurrent with the experiments, but done posteriorly. In those cases, we ensured similar culture growth conditions; a posteriori comparison with previously measured CNP contents from our culture collection revealed the suitability of elemental and stoichiometric data obtained. For the mixotrophic and heterotrophic protists, when small amounts of their own cryptophyte prey had remained at the time of preparing the experimental suspensions, their contribution to the elemental content of the copepod prey was subtracted after analysis. Stoichiometric ratios were calculated as molar ratios and propagation of error was taken into account due to independency between CN and P samples^68^.

### Statistical analysis

Graphics and statistical analyses were conducted with the software SigmaPlot 14.0. Data were checked for normality (Shapiro–Wilk) and homoscedasticity (Brown-Forsythe). One-way ANOVA and post-hoc comparisons (Tukey HSD, Bonferroni tests) were performed to assess differences in the copepod physiological rates among functional trophic modes. Kruskal–Wallis and Dunn’s post hoc test were performed instead of ANOVA when homoscedasticity assumptions were not met. More specifically, we compared copepod physiological responses across all the diets under the three functional trophic modes (auto-, mixo-, heterotrophic); we did the same kind of analysis also independently for the specific *K. veneficum* comparisons (autotrophic in f/2, autotrophic in FSW and mixotrophic in FSW + *R. salina*). In addition, we tested for differences of copepod ingestion rate in relation to prey size classes applying the same procedure as with functional trophic modes. The size classes, small (7–13 µm), intermediate (13–18 µm) and large (18–31 µm), were established on the basis of optimal prey:predator ratio from previous works and on the likely occurrence of sloppy feeding^4^. Results were considered significant at the 0.05 level. Generally, in case of non-significant comparisons, only *P* values from ANOVA and Kruskal–Wallis tests are reported; on the contrary, if those tests yielded significant differences (by default *P* < 0.05), only *P* values relative to the post-hoc analysis are reported. Simple linear regression analyses were conducted between copepod physiological responses and prey stoichiometric traits as proxies of food quality (one-way ANOVA output reported). Graphical exploration of morpho-stoichiometric characteristics of prey species and clustering of prey trophic modes was performed with a multivariate Principal Component Analysis (PCA).

## Supplementary information

Supplementary Table 1.

## Data Availability

Requests regarding the dataset generated during the current study should be addressed to C.T.
