# Supplementary material for: Effects of prey trophic mode on the gross-growth efficiency of marine copepods: the case of mixoplankton
Source: Sci Rep. 2020 Jul 23;10:12259. doi: 10.1038/s41598-020-69174-w (PMC7378051; doi:10.1038/s41598-020-69174-w)
Supplement: Supplementary file 1 — Supplementary Table 1. [file 41598_2020_69174_MOESM1_ESM.pdf]

Effects of prey trophic mode on the gross-growth efficiency of marine copepods: the case of mixoplankton

Claudia Traboni[1,2*], Albert Calbet[1], Enric Saiz[1]

Table S1. Statistical outputs of simple linear regressions testing the relationship between copepod physiological responses versus the prey stoichiometric ratios as proxies of food quality. Significant values are indicated with *. [1]Notice that when an outlier (*M. rubrum*) was removed from the analysis, the hatching success vs C:P regression resulted to be non-significant (P = 0.233).

Physiological response	Prey stoichiometric ratio					
	C:N		C:P		N:P	
	R^2	P	R^2	P	R^2	P
Ingestion rate	0.0115	0.727	0.177	0.153	0.314	0.046*
Egestion rate	0.0139	0.701	0.0474	0.475	0.140	0.207
E/I ratio	0.0316	0.561	0.0449	0.487	0.206	0.120
Egg production rate	0.0159	0.681	0.130	0.225	0.0904	0.318
Gross-growth efficiency	0.00009	0.975	0.168	0.164	0.217	0.109
Hatching success	0.0417	0.503	0.324	0.042*,[1]	0.245	0.085
